# Synthesis and Characterization of New Schiff Bases Derived from N (1)-Substituted Isatin with Dithiooxamide and Their Co(II), Ni(II), Cu(II), Pd(II), and Pt(IV) Complexes

**DOI:** 10.1155/2009/413175

**Published:** 2009-10-26

**Authors:** Ahlam J. Abdul-Ghani, Asmaa M. N. Khaleel

**Affiliations:** Department of Chemistry, College of Science, University of Baghdad, Jaderiya, P.O. Box 47059 Baghdad, Iraq

## Abstract

Three new Schiff bases of N-substituted isatin L_I_, L_II,_ and L_III_ = Schiff base of N-acetylisatin, N-benzylisatin, and N-benzoylisatin, respectively, and their metal complexes C_1a,b_ = [Co_2_(L_I_)_2_Cl_3_]Cl, C_2_ = [Ni(L_I_)_2_Cl_2_]0.4BuOH, C_3_ = [CuL_I_Cl(H_2_O)]Cl ⋅ 0.5BuOH, C_4_ = [Pd(L_I_)_2_Cl]Cl, C_5_ = [Pt(L_1_)_2_Cl_2_]Cl_2_ ⋅ 1.8EtOH.H_2_O, C_6a_ = [CoL_II_Cl]Cl ⋅ 0.4H_2_O ⋅ 0.3DMSO, C_6b_ = [CoL_II_Cl]Cl ⋅ 0.3H_2_O ⋅ 0.1BuOH, C_7_ = [NiL_II_Cl_2_], C_8_ = [CuL_II_]Cl_2_ ⋅ H_2_O* *, C_9_ = [Pd(L_II_)_2_]Cl_2_, C_10_ = [Pt(L_II_)_2.5_Cl]Cl_3_, C_11a_ = [Co(L_III_)]C_12_ ⋅ H_2_O, C_11b_ = [Co(L_III_)]Cl_2_ ⋅ 0.2H_2_O, and C_12_ = [Ni(L_III_)_2_]Cl_2_, C_13_ = [Ni(L_III_)_2_]Cl_2_ were reported. The complexes were characterized by elemental analyses, metal and chloride content, spectroscopic methods, magnetic moments, conductivity measurements, and thermal studies. Some of these compounds were tested as antibacterial and antifungal agents against *Staphylococcus aureus, Proteus vulgaris, Candida albicans, and Aspergillus niger*.

## 1. Introduction

**Scheme 1:**
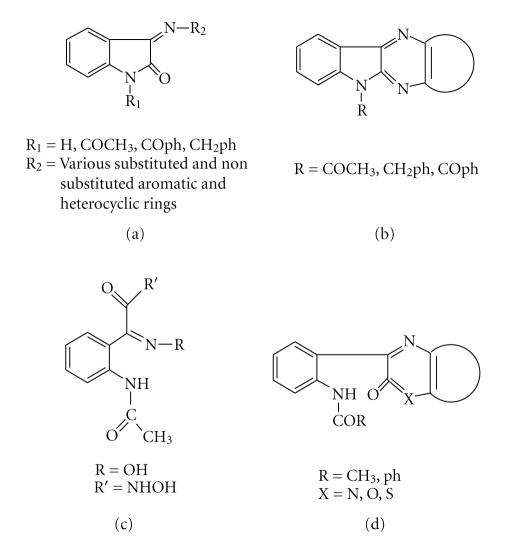
Schiff bases of isatin derivatives.

Isatin (indole-2,3-dione) and its derivatives have shown a wide scale of biological activities such as antibacterial [1–3], antifungal [1, 3–5], anticonvulsant [2, 6], anti-HIV [7], anticancer [1, 2], antiviral [1], and enzyme inhibitors [2]. The Schiff bases (a) and (b) (Scheme 1) derived from isatin and its derivatives with different amines have been studied [1, 2, 6, 8–13]. The reaction of N-acetyl, N-benzoyl, and N-tosylisatin and their Schiff base derivatives (c) and (d) (Scheme 1) with ethanol, methanol, isopropyl alcohol, allyl alcohol, TsNH_2_, pyrrolidine, and water yield products resulting from nucleophilic attack at the C-2 carbonyl that leads to heterocyclic ring cleavage [8, 14]. The present work aims to study the synthesis and antibacterial activity of three new ligands derived from condensation of N-acetyl, N-benzyl, and N-benzoylisatin with the chelating agent dithiooxamide (ethanedithioamide or rubeanic acid) *dto* and their metal complexes. The Schiff bases of dithiooxamide and their complexes have received most of the attention because of the semiconductive, magnetic, spectroscopic, and thermal properties [15–17] as well as being used as semiconductors antibacterial and antifungal agents [18–20].

## 2. Experimental/Materials and Methods

All chemicals used were of analytical reagent grade (AR) except *dto* and ethanol which were purified prior to use [21]. FTIR spectra were recorded on SHIMADZU FTIR-8400S, Fourier Transform, Infrared spectrophotometer. The electronic spectra (*λ*(200–1100) nm) in different solvents were recorded on Shimadzu (UV-Vis)-160 spectrophotometer. Elemental microanalyses were performed on Euro vector EA 3000 A. The metal contents of the complexes were determined by atomic absorption technique using Varian-AA775, Atomic Absorption Spectrophotomer. Mass spectra were recorded on Shimadzu QP 5050A. ^1^H NMR was performed by using Bruker Ultra Sheild 300 MHz NMR spectrophotometer. Thermal analyses (TG and DTG) were carried out by using Shimadzu Thermal Analyzer Type 50 H. Electrical conductivity measurements for complexes (10^−3^ M) in DMF and DMSO at room temperature were carried out by using Hunts Capacitors Trade Mark British made. Magnetic moments (*μ*_eff._ B.M) for the prepared complexes in the solid state at room temperature were measured by using Bruker Magnet B.M-6. The chloride content for complexes was determined by Mohr's method. N-acetylisatin, N-benzylisatin, N-benzoylisatin, and PdCl_2_(phCN)_2_ were prepared by methods reported in literature [6, 22–25].

## 3. Synthesis of Ligands

**Scheme 2:**
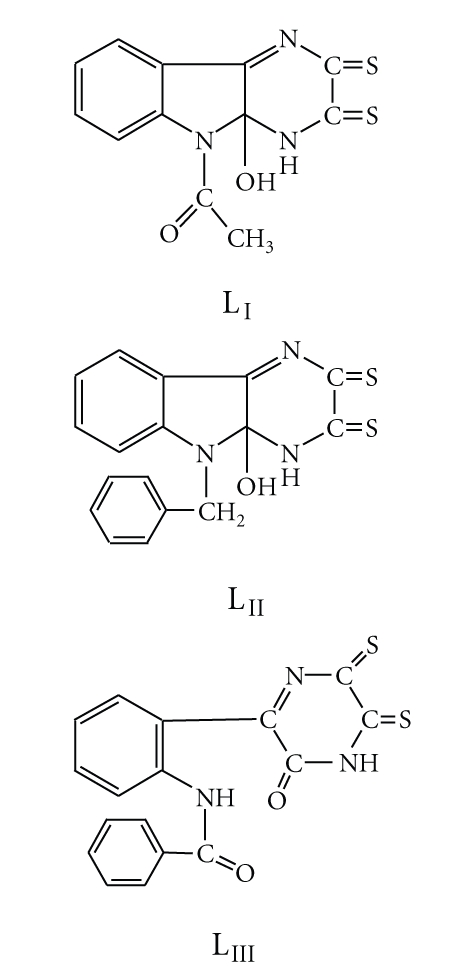
The structures of the prepared ligands L_I_, L_II_, and L_III_.

All attempts to prepare 1-(9a-Hydroxy-2,3-dithiooxo-1,2,3,9a-tetrahydro-1,4,9-triaza-fluoren-9-yl)-ethanone (L_I_) (Scheme 2) and 9-Benzyl-9a-hydroxy-9,9a-dihydro-1H-1,4,4-triaza-fluorene-2,3-dithione (L_II_) (Scheme 2) in solutions were unsuccessful; therefore solid reaction was carried out to prepare the two ligands.

### 3.1. Schiff Base of N-Acetylisatin: 1-(9a-Hydroxy-2,3-dithiooxo-1,2,3,9a-tetrahydro-1,4,9-triaza-fluoren-9-yl)-ethanone (L_I_)

A powdered mixture of N-acetylisatin (0.3092 g, 1.6 mmol) and *dto* (0.0983 g, 0.8 mmol) in a sealed Carius tube was heated in a stirred oil bath at 160–170°C for 2 hours. The melt colour was changed from orange to dark brown. After cooling to room temperature, the solid product was ground and dissolved in butanol, followed by precipitation with ether. A black precipitate was formed. The product was filtered off and washed several times with ether to remove the unreacted materials giving brown crystals. Yield (0.116 g, 48.76%), m.p (220°C decomp.). ^1^H NMR data *δ*(ppm), (CDCL_3_): 2.508 (3H,s,CH_3_); 3.34 (2H,s,OH and NH thioamide); 7.074–7.853 (4H, m, aromatic protons). MS(EI), m/z(%): 207(21), 161(10), 146(23), 133(9), 92(10), 78(59), 63(84), 44(100). Anal. for C_12_H_9_N_3_O_2_S_2_ Calcd. C, 49.48; H, 3.09; N, 14.43%; Found: C, 50.54; H, 3.22; N, 13.23%.

### 3.2. Schiff Base of N-Benzylisatin: 9-Benzyl-9a-hydroxy-9,9a-dihydro-1H-1,4,4-triaza-fluorene-2,3-dithione (L_II_)

A powdered mixture of N-benzylisatin (0.829 g, 3.5 mmol) and *dto* (0.85 g, 7 mmol) was heated in a sealed Carius tube in an oil bath at 140°C for 10 hours. Colour of melt was changed from orange to dark brown. After cooling to room temperature, a solid mass was formed. The product was ground and purified several times in refluxing ethanol, filtered off, washed with hot ethanol followed by acetone and dried, giving dark brown crystals. Yield (0.2679 g, 22.3%), m.p (>250°C). ^1^H NMR data *δ*(ppm), (DMSO): 3.45(2H, s, OH and NH thioamide); 5.1(2H, w, CH_2_ benzyl); 6.9–7.2(9H, m, aromatic protons). MS(EI), m/z(%): 156(11), 149(15), 127(13), 105(11), 78(100), 63(100). Anal. for C_17_H_13_N_3_OS_2_ Calcd.: C, 60.17; H, 3.83; N, 12.38%; Found: C, 61.10; H, 3.43; N, 12.98%.

### 3.3. Schiff Base of N-Benzoylisatin: N-[2-(3-oxo-5,6-dithioxo-3,4,5,6-tetrahydro-pyrazin-2-yl)-phenyl] benzamide (L_III_)

Equimolar amounts of benzoylisatin (0.2 g, 0.79 mmol) and *dto* (0.0957 g, 0.79 mmol) in butanol (2 cm^3^) containing 4 drops of piperidine were heated under reflux with stirring for 5 hours during which the colour of solution was changed from orange to brown. The solution mixture was left to stand overnight and then cooled down to 0°C. Cold ether was added until a dark brown precipitate was formed. The product was filtered off, washed several times with acetone followed by ether. Yield (0.0857 g, 30.51%), m.p. (250°C decomp.), ^1^H NMR data (ppm), (DMSO): 4.902–5.101(1H, b, NH thioamide); 7.144–7.860(9H, m, aromatic protons); 10.124(1H, b, NH benzoyl moiety). MS(EI), m/z(%): 296.6(13), 267.5(7), 232.6(20), 195.6(15), 149.5(6), 104.4(27), 83.4(6). Anal. for C_17_H_11_N_3_O_2_S_2_ Calcd.: C, 57.79; H, 3.11; N, 11.89%; Found: C, 57.39; H, 3.54; N, 11.30%.

## 4. Preparation of Metal Complexes

- A solution mixture of the ligands L_I_ and L_II_ (0.01 mmol) (0.0029, 0.0033 g), respectively, with the metal salts CoCl_2_ ⋅ 6H_2_O, NiCl_2_ ⋅ 6H_2_O, and CuCl_2_ ⋅ 2H_2_O (0.01 mmol) and (0.02 mmol) (0.0058, 0.0067 g) of L_I_ and L_II_, respectively, with the metal salts PdCl_2_(phCN)_2_ and K_2_PtCl_6_ (0.01 mmol), in DMF (C_1_), butanol (C_2_ and C_3_), or DMSO (C_4_–C_10_) was heated under reflux for four hours. Precipitation of L_I_ complexes took place within 30 minutes, while those of L_II_ was precipitated at the end of reflux time. The products were filtered, washed with hot ethanol and acetone, followed by ether and vacuum dried. Complexes of L_III_ were prepared in the same manner using a mixture of L_III_ (0.01 mmol, 0.0035 g) with the metal salts CoCl_2_ ⋅ 6H_2_O, NiCl_2_ ⋅ 6H_2_O (0.01 mmol), and (0.02 mmol, 0.007 g) of L_III_ with PdCl_2_(phCN)_2_ (0.01 mmol). “C_1a_”: colour(dark brown) Yield (26.24%). Anal. for (C_24_H_18_N_6_O_4_S_4 _Co_2_Cl_3_)Cl Calcd.: C, 34.21; H, 2.13; N, 9.97; S, 15.20%; Found: C, 34.32; H, 2.50; N, 9.42; S, 15.13%. M, 13.99(Calcd), 14.0(Found)%; Cl, 16.86(Calcd), 16.20(Found)%. “C_2_”: colour(dark brown). Yield (23.51%). Anal. for [(C_24_H_18_N_6_O_4_S_4 _NiCl_2_)0.4(C_4_H_10_O)] Calcd.: C, 47.63; H, 5.75; N, 8.33%; Found: C, 48.32; H, 5.45; N, 8.83%. M, 5.82(Calcd), 5.72(Found)%. “C_3_”: colour(dark brown). Yield (23.35%). Anal. for [(C_12_H_9_N_3_O_2_S_2_CuCl (H_2_O))Cl.0.5(C_4_H_10_O)] Calcd.: C, 34.96; H, 3.32; N, 8.74%; Found: C, 34.01; H, 3.72; N, 9.73%. M, 13.21(Calcd), 13.85(Found)%. Cl, 14.77(Calcd), 14.70(Found%). “C_4_”: colour(dark brown). Yield (35.18%). Anal. for [(C_24_H_18_N_6_O_4_S_4_PdCl)Cl] Calcd.: C, 37.94; H, 2.37; N, 11.06; S, 16.86%; Found: C, 38.40; H, 2.38; N, 11.15; S, 16.78%. M, 13.96(Calcd), 13.71(Found)%. Cl, 9.35(Calcd), 10.5(Found)%. “C_5_”: colour(brown). yield (19.08%). Anal. for [(C_24_H_18_N_6_O_4_S_4_PtCl_2_)Cl_2_ ⋅ 1.8(C_2_H_6_O) ⋅ H_2_O] Calcd.: C, 32.47; H, 3.02; N, 8.23%; Found: C, 32.85; H, 3.27; N, 9.16%. M, 19.12(Calcd), 19.10(Found)%. “C_6a_”: colour(dark brown). Yield (36.06%). Anal. for [(C_17_H_13_N_3_OS_2_CoCl)Cl ⋅ 0.4(H_2_O) ⋅ 0.3(C_2_H_6_SO)] Calcd.: C, 42.28; H, 3.12; N, 8.40; S, 12.81%; Found: C, 41.85; H, 2.72; N, 8.15; S, 12.00%. M, 11.79(Calcd), 12.11(Found)%; Cl, 14.21(Calcd), 14.58(Found)%. “C_7_”: colour(dark brown). Yield (45.30%). Anal. for [C_17_H_13_N_3_OS_2_NiCl_2_] Calcd.: C, 43.52; H, 2.77; N, 8.96%; Found: C, 44.20; H, 3.05; N, 9.24%. M, 12.52(Calcd), 12.23(Found)%; Cl, 15.14(Calcd), 15.47(Found)%. “C_8_”: colour(dark brown). Yield (45.39%). Anal. for [(C_17_H_13_N_3_OS_2_Cu)Cl_2_ ⋅ H_2_O)] Calcd.: C, 41.50; H, 3.05; N, 8.54%; S, 13.02%; Found: C, 42.15; H, 2.74; N, 8.16; S, 12.94%. M, 12.91(Calcd), 13.12(Found)%; Cl, 14.44(Calcd), 14.55(Found)%. “C_9_”: colour(dark brown). Yield (43.38%). Anal. for [(C_34_H_26_N_6_O_2_S_4_Pd)Cl_2_)] Calcd.: C, 47.71; H, 3.04; N, 9.82%; S, 14.97%; Found: C, 47.75; H, 3.11; N, 9.69; S, 15.52%. M, 12.39(Calcd), 13.00(Found)%; Cl, 8.30(Calcd), 8.47(Found)%. “C_10_”: colour(dark brown). Yield (32.42%). Anal. for [((C_17_H_13_N_3_OS_2_)_2.5_PtCl)Cl_3_] Calcd.: C, 43.05; H, 2.74; N, 8.86%; S, 13.50%; Found: C, 43.84; H, 2.55; N, 9.17; S, 14.09%. M, 16.46(Calcd), 15.87(Found)%. “C_11a_”: colour(brown). Yield (33.43%). Anal. for [(C_17_H_11_N_3_O_2_S_2_Co)Cl_2_ ⋅ H_2_O] Calcd.: C, 40.72; H, 2.59; N, 8.38 %; Found: C, 40.05; H, 2.34; N, 7.82;%. M, 11.75(Calcd), 12.25(Found)%. Cl, 14.17(Calcd), 14.85(Found)%. “C_12_”: colour(brown). Yield (34.37%). Anal. for [(C_34_H_22_N_6_O_4_S_4_Ni)Cl_2_] Calcd.: C, 48.82; H, 2.63; N, 10.05%; Found: C, 49.62; H, 2.46; N, 9.41; %. M, 7.02(Calcd), 7.50(Found)%. Cl, 8.49(Calcd), 8.56(Found)%. “C_13_”: colour(brown). Yield (34.03%). Anal. for [(C_34_H_22_N_6_O_4_S_4_Pd)Cl_2_] Calcd.: C, 46.20; H, 2.46; N, 9.51; S, 14.49%; Found: C, 46.47; H, 2.66; N, 9.79; S, 14.78%. M, 12.00(Calcd), 11.50(Found)%. Cl, 8.04(Calcd), 8.63(Found)%.
- To a solution mixture of N-acetyl, N-benzyl, or N-benzoylisatin (0.02 mmol) 0.0037, 0.0047, and 0.005 g, respectively, with *dto* (0.01 mmol) (0.0012 g), (0.04 mmol) (0.0048 g), and (0.02 mmol) (0.0024 g), respectively, in butanol was added a solution of CoCl_2_ ⋅ 6H_2_O (0.02 mmol) in butanol. The mixture was heated under reflux. Precipitation took place immediately. Heating was continued for 4 hours to achieve complet precipitation. The product was filtered, washed with hot butanol, followed by ethanol, acetone, ether, and vacuum dried. “C_1b_”: colour(dark brown). Yield (25.15%). Anal. for (C_24_H_18_N_6_O_4_S_4 _Co_2_Cl_3_)Cl Calcd.: C, 34.21; H, 2.13; N, 9.97; S, 15.20%; Found: C, 34.15; H, 1.95; N, 10.31; S, 15.07%. M, 13.99(Calcd), 13.30(Found)%; Cl, 16.86(Calcd), 16.19(Found)%. “C_6b_”: colour(dark brown). Yield (71.42%). Anal. for [(C_17_H_13_N_3_OS_2_CoCl) Cl ⋅ 0.3(H_2_O) ⋅ 0.1(C_4_H_10_O)] Calcd.: C, 43.34; H, 3.03; N, 8.71; S, 13.28%; Found: C, 43.89; H, 3.35; N, 8.54; S, 13.48%. M, 12.22(Calcd), 12.25(Found)%; Cl, 14.73(Calcd), 14.44(Found)%. “C_11b_”: colour(brown). Yield (41.72%). Anal. for [(C_17_H_11_N_3_O_2_S_2_Co)Cl_2_ ⋅ 0.2H_2_O] Calcd.: C, 41.93; H, 2.34; N, 8.63%; Found: C, 42.56; H, 2.53; N, 8.73; %. M, 12.10(Calcd), 12.11(Found)%. Cl, 14.59(Calcd), 14.71(Found)%.

## 5. Microbiological Test Methods

The two following methods were used to perform the antimicrobial tests.

### 5.1. Agar Diffusion Method

In this method the colonies of the selected bacteria, namely, *Staphylococcus aureus* (G^+^), *Proteus vulgaris* (G^−^), and the fungus *Candida albicans* were spread on the surface of solidified nutrient agar. Suitably separated 7 mm diameter holes were made in each agar plate. Each hole was injected with 0.1 mL of 150, 350, 650, and 1000 ppm of the studied compound in DMSO. The agar plates were incubated at 37°C for 24 hours. Diameters of growth inhibition zones were measured in mm depending on diameter and clarity.

### 5.2. Agar Dilution Method

In this method the antifungal activity of 250 ppm of some selected compounds in DMSO was screened against *Aspergillus niger.* 2.5 cm^3^ of 2000 ppm of tested solution was added to 20 cm^3^ of hot agar solution. The homogenized mixture was then poured into petridish and left to solidify. The *Aspergillus *colony (9 mm diameter) was fixed on the solidified agar, and the medium was incubated at 37°C for 8 days.

## 6. Results and Discussion

The IR spectra showed that the three ligands exhibited vibrational modes of *ν*_C=N_ of azomethine group [4, 6, 26–28], (*ν*_C–N_, *δ*_NH_), (*ν*_C–N_, *ν*_C–S_), *ν*_C–S_, and *ν*_C=S_ of *dto* moiety [29, 30] (Table 1). Spectra of L_I_ and L_II_ showed vibrational bands related to stretching modes of OH groups [31, 32]. The position of the bands assigned to *ν*_NH_ vibrations of the cyclic rings was dependent on their environment. *ν*_NH_ of L_II_ and L_III_ were observed at lower frequencies compared with that of L_I_ (Table 1) [27, 32]. The latter exhibited bands assigned to *ν*_C=O_ and *ν*_NH_ of amide and lactam rings [6, 27, 31, 32]. The spectra of L_I_ complexes with Co(II), Cu(II), and Pd(II) ions exhibited shift in *ν*_OH_ and *ν*_C=N_ (azomethine) vibrations. The latter two complexes together with Ni(II) complex showed additional shifts in *ν*_NH_ to lower frequencies while no significant changes were observed on vibrational modes of C=O group which rules out coordination with carbonyl oxygen. Shifts of thioamide bands (III and IV) were observed in the spectra of Cu(II) and Pt(IV) complexes and were attributed to coordination of metal ion with sulfur atom [33]. Metal complexes of L_II_ showed bands assigned to *ν*_C=O_ and *ν*_NH2_ vibrations (Table 1). This may be attributed to cleavage of thioamide ring on complexation leading reappearance of *ν*_C=O_ and *ν*_NH2_ of both C-2 and NH_2_ of isatin and *dto* moieties, respectively. Shifts in *ν*_NH2_ (compared with *ν*_NH2_ of the free *dto* (3296, 3203 cm^−1^)) [34] to lower frequencies were observed in all spectra of complexes except that of Ni(II) which was shifted to higher frequency. Bands related to *ν*_C=O_ vibrations in spectra of both Ni(II) and Cu(II) complexes were shifted to higher frequencies while spectra of the other complexes showed shifts to lower frequencies. Additional shifts were observed in the bands assigned to *ν*_C=N_ (azomethine) in all complexes except that of Cu(II). The latter complex exhibited shift of *ν*_C=S_ band to lower frequency which refers to coordination of sulfur to Cu(II) ion [33]. The spectra of L_III_ metal complexes exhibited shifts in vibrational modes of *ν*_C=O_ and band IV of thioamide group as a result of coordination with metal ions [33, 35]. Additional shift in position of bands assigned to *ν*_C=N_ was observed in the spectra of Co(II) and Ni(II) complexes. Shifts in the position of *ν*_NH_ amide and *ν*_C=O_ of lactam ring were observed in the spectra of the Pd(II) complex as a result of coordination. Bands related to vibrational modes of lattice solvent, coordinated water were observed at 3500-3400 cm^−1 ^ [36–38]. Bands appeared at lower frequencies were refered to M–O, M–N, M–S, and M–Cl stretching modes [36–38]. Further data are collected in (Table 1).

**Scheme 3:**
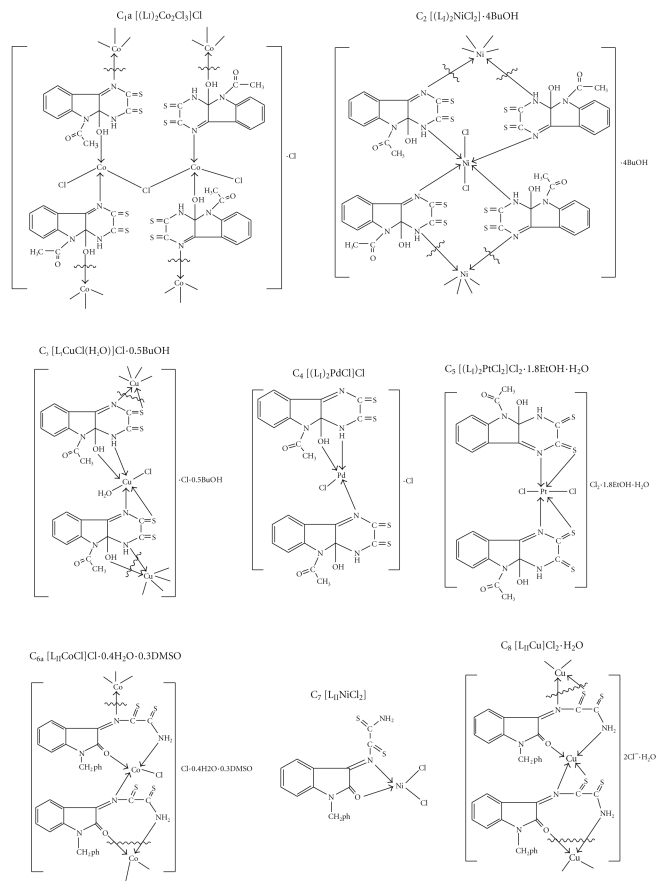

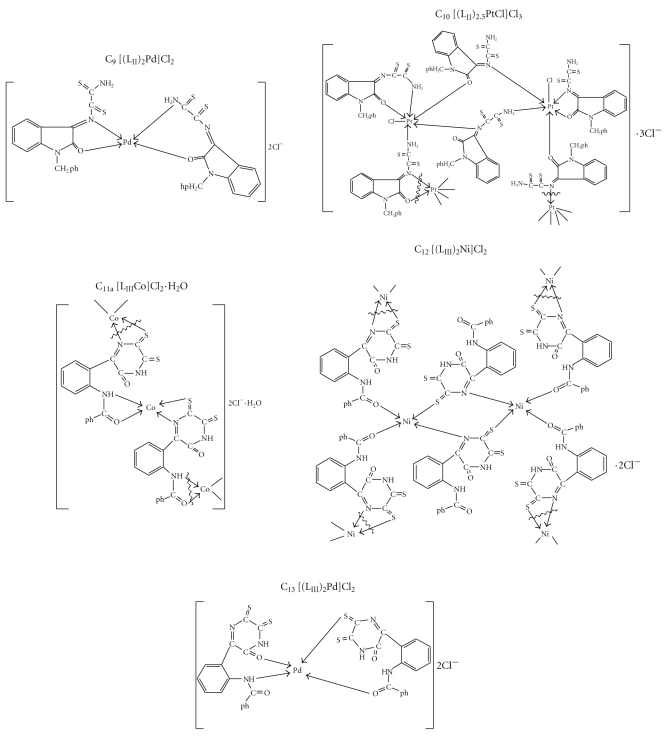
Suggested structures of studied compounds.

The electronic spectra of L_I_, L_II_, and L_III_ exhibited high-intensity multiple bands in DMF and DMSO at 36231–20000 cm^−1^. These bands were assigned to *π* → π* transition of conjugated system. L_III_ exhibited additional low-intensity band which was assigned to n → π* transition. Changes in positions and profile (C_8_–C_10_) of bands were observed in the spectra of metal complexes. Bands related to the (CT) transition were observed as a shoulder on the ligand band in the spectra of C_1_, C_3_, C_6_, C_7_, C_9_, and C_10_ complexes (Table 2). The bands observed in the spectra of Co(II) complexes in the visible region were assigned to ^4^A_2_ → ^4^T_2_(*ν*_1_), ^4^A_2_ → ^4^T_1_(F)(*ν*_2_), and ^4^A_2_ → ^4^T_1_(P)(*ν*_3_). The magnetic moment values of Co(II) complexes were in the range of (3.959–4.6 BM) (Table 2). This indicates tetrahedral geometry around Co(II) ions [36–39] (Scheme 3). The Ni(II) complex C_2_ gave a greenish yellow colour in DMF indicating the exchange of weak ligand atoms with solvent molecules [40–43]. The spectrum of this complex showed bands characteristic of octahedral Ni(II) complex [36–38, 40–43] (Table 2), while the other Ni(II) complexes (C_7_ and C_12_) showed tetrahedral geometries (Scheme 3).

The electronic spectra and magnetic moments (*μ*_eff_ B.M) (Table 2) of these complexes were consistent with these assignment [36–38, 40–43]. Spectral data (B′, Dq/B′, 10Dq and *β*) (Table 2), for the Co(II) and Ni(II) complexes were calculated by applying band energies on Tanaba Saugano diagrams. The energy of *ν*_1_ for Co(II) complexes (C_1_, C_6_, C_11_) and Ni(II) complexes (C_7_, C_12_) and *ν*_3_ for Ni(II) complex C_2_ were also calculated from the diagrams. The spectrum of the Cu(II) complex C_3_ exhibited three bands (Table 2) attributed to the spin allowed transitions ^2^B_1_g → ^2^A_1_g(*ν*_1_), ^2^B_1_g → ^2^B_2_g(*ν*_2_) and ^2^B_1_g → ^2^Eg(*ν*_3_) of Jahn Teller tetragonally distorted octahedral Cu(II) complexes [34]. The magnetic moment of the complex (2.36 B.M) indicated paramagnetic character with a high spin orbital coupling [40–43]. The spectrum of Cu(II) complex C_8_ exhibited two bands (Table 2) which were assigned to ^2^B_1_g → ^2^A_1_g(*ν*_1_), and ^2^B_1_g → ^2^B_2_g(*ν*_2_). These bands were attributed to square planar Cu(II) complexes [44] (Scheme 3). Magnetic moment (*μ*_eff_ = 1.84 B.M) of the complex supported such conclusion [36–38, 44]. The spectra of the diamagnetic Pd(II) complexes (C_4_, C_9_, and C_13_) showed two bands assigned to ^1^A_1_g → ^1^A_2_g(*ν*_1_) and ^1^A_1_g → ^1^B_1_g(*ν*_2_) and the additional band ^1^A_1_g → ^1^Eg(*ν*_3_) for C_4_. These bands are attributed to square planar Pd(II) complexes [34–38, 40–43]. The spectra of the diamagnetic Pt(IV) complexes exhibited two bands which were assigned to forbidden transitions ^1^A_1_g → ^3^T_1_g and ^1^A_1_g → ^3^T_2_g showing octahedral geometry around Pt(IV) ion [40–43] (Scheme 3). The molar conductivities (Table 2) showed that electrolytic nature of the Pt(IV) complex (C_10_) was 1 : 3, Pt(IV), Cu(II), Pd(II), Co(II) and Ni(II) complexes (C_5_, C_8_, C_9_, C_11_, C_12_, and C_13_) 1 : 2, and Co(II), Cu(II), and Pd(II) complexes (C_1_, C_3_, C_4_, and C_6_) 1 : 1, while the Ni(II) complexes (C_2_ and C_7_) were nonelectrolyte [45]. From these observations, together with the results obtained from other analytical data, the sterochemical structures of the complexes were suggested (Scheme 3).

Thermogravimetric analyses (TG and DTG) have been studied at heating range of 50–800°C for the complexes (C_1_, C_3_, C_4_, and C_7_) under nitrogen atmosphere. The following results (Table 3) were explained according to analytical suggestions mentioned in literature [46–48]. (i) Lattice water, free ions, and organic fragments that are not directly coordinated to the metal ions were found to leave the complex at earlier stages compared with coordinated fragments, (ii) The heating range (50–800°C) produced incomplete decomposition of metal complexes, and the final products were dependent on the type of metal ion and on (M-L) affinity [36–38, 46, 49] which reflects the stability of complexes.

## 7. Biological Screening

The antibacterial activity for precursors, L_I_ and L_III_, and some of their complexes was evaluated against *Staphylococcus aureus* (G^+^) and *Proteus vulgaris* (G^−^) using the agar diffusion method. Diameter (mm) of growth inhibition zones was measured after incubation for 24 hours at 37°C. The results showed that no antibacterial action was recorded by the studied compounds using concentration of 150, 350, and 650 ppm. Using 1000 ppm (Table 4), L_I_ and its complexes were more active against *Staphylococcus aureus*, while L_III_ and its complexes (except C_13_) were more active against *Proteus vulgaris* than the other studied compounds. The antifungal activity was evaluated against *Candida albicans* by the agar diffusion method and *Aspergillus niger* colony (9 mm diameter) by the agar dilution method using concentration of 250 ppm in DMSO. The results showed that L_I_ and L_III_ were inactive against *Candida albicans*; Co(II) (C_11_), Ni(II) (C_12_), and Pd(II) (C_13_) complexes were more active than the parent ligand (L_III_) while those of L_I_ were inactive except Cu(II) complex (C_3_). L_I_, L_III_, and C_4_ which were inactive against *Candida albicans* showed moderate activity against *Aspergillus niger* which refer to the effective selectivity of specific inhibitor on the microorganisms.

## 8. Conclusions

1. Condensation reaction of N-acetyl, N-benzyl, and N-benzoyl isatins with *dto* gave Schiff base ligands L_I_–L_III_, as was confirmed by ^1^H, ^13^C NMR, and IR spectra.
2. The formation of the Schiff base ligand L_III_ took place with ring cleavage at C-2 of the heterocyclic ring of the benzoylisatin. Whereas the formation of L_I_ and L_II_ took place without ring cleavage.
3. The presence of various donor atoms and the stereochemistry of the studied ligands enhanced different complexing behaviours and geometries using the studied metal ions.
4. The results of the physical properties and spectral analyses of cobalt complexes prepared by template reaction demonstrated the recommendation of for synthesis of metal complexes of the studied ligands, due to less time consuming and in general more yield of products.
5. The study of biological activity of the studied ligands and some of their metal complexes against bacteria and fungi showed selectivity nature of microorganism towards these compounds and indicated the possibility of using some of them as antibacterial and antifungal agents.

## Figures and Tables

**(a):** 

Symbol	*ν*_OH_	*ν*_N–H_	*ν*_C=O_	*ν*_C=N_	Thioamide				*ν*_M–O_	*ν*_M–N_	*ν*_M–Cl_
					Band I	Band II	Band III	Band IV			
					*ν*_C–N_ + *δ*_NH_	*ν*_C–N_ + *ν*_C–S_	*ν*_C–S_	*ν*_C=S_			
L_I_	3400	3298	1710	1650	1540	1465	1170	881	—	—	—
C_1a_	3344	3295	1718	1631	1545	1460	1162	877	559	389	277*
Co(II)											
C_1b_	3350	3295	1718	1631	1545	1460	1165	877	559	389	277*
Co(II)											
C_2_	3402	3227	1706	1631	1540	1396	1165	880	—	335	320
Ni(II)											
C_3_	3347	3260	1716	1627	1520	1450	1150	780	408	350	339
Cu(II)											
C_4_	3395	3250	1720	1630	1573	1458	1170	889	586	350	331
Pd(II)											
C_5_	3400	3295	1715	1666	1510	1483	1134	850	—	340	300
Pt(IV)											

Lattice butanol, C_2_, C_3 _= 3500, 3750 cm^−1^, Lattice ethanol, C_5_ = 3495 cm^−1^ Coord ⋅ H_2_O, C_3_ = 3456, 750, 675; Lattice H_2_O, C_5_ = 3425 cm^−1^ *ν*_M-S_, C_3_ and C_5_ = 345 and 370 cm^−1^ respectively, *bridging.

**(b):** 

Symbol	*ν*_NH_2__	*ν*_C=O_	*ν*_C=N_	Thioamide				H_2_O Lattice	*ν*_M-O_	*ν*_M-N_	*ν*_M-Cl_
				Band I	Band II	Band III	Band IV				
				*ν*_C–N_ + *δ*_NH_	*ν*_C–N_ + *ν*_C–S_	*ν*_C–S_	*ν*_C=S_				
L_II_	—	—	1604	1554	1461	1170	848	—	—	—	—
C_6a_	3255	1724	1612	1555	1446	1165	868	3417	547	466	273
Co(II)	3160									401	
C_6b_	3250	1720	1612	1560	1450	1180	864	3450	493	450	230
Co(II)	3155									385	
C_7_	3450	1750	1620	1550	1448	1150	870	—	520	400	316
Ni(II)	3348										
C_8_	3224	1751	1589	1548	1448	1188	817	3450	560	478	—
Cu(II)	3132									385	
C_9_	3147	1720	1612	1550	1465	1180	856	—	500	400	—
Pd(II)	3047									380	
C_10_	3294	1720	1612	1548	1472	1170	850	—	500	400	330
Pt(IV)	3147									370	

*ν*_OH,_ L_II_ = 3400 cm^−1^; *ν*_NH_, L_II_ = 3145 cm^−1^; Lattice butanol, C_6b_ = 3550 cm^−1^ *ν*_M-S_, C_8_ = 320 cm^−1^.

**(c):** 

Symbol	*ν*_N–H_ amide	*ν*_C=O_ amide	*ν*_N–H_ lactam	*ν*_C=O_ lactam	*ν*_*c*=*N*_	Thioamide group				*ν*_M–O_	*ν*_M–N_	*ν*_M–S_
						Band I	Band II	Band III	Band IV			
						*ν*_C–N_ + *δ*_NH_	*ν*_C–N_ + *ν*_C–S_	*ν*_C–S_	*ν*_C=S_			
L_III_	3394	1635	3247	1674	1600	1535	1465	1103	880	—	—	—
C_11a_	3410	1625	3247	1674	1587	1535	1450	1095	830	590	480	320
Co(II)												
C_11b_	3410	1620	3247	1674	1580	1535	1450	1100	840	600	480	300
Co(II)												
C_12_	3456	1625	3250	1670	1589	1535	1450	1100	860	580	450	308
Ni(II)												
C_13_	3386	1620	3250	1666	1600	1535	1473	1095	850	617	401	310
Pd(II)												

Lattic H_2_O, C_11a_, C_11b_ = 3500 cm^−1^

**Table 2:** Electronic spectra, spectral parameters and magnetic moment with suggested structures of L_I_, L_II_, and L_III_ complexes.

Symbol	Band positions (cm^−1^)	Assignment	Dq/$\bar{\text{B}}$ (*β*)	$\bar{\text{B}}$ (cm^−1^)	10Dq (cm^−1^)	*μ*_eff_ (B.M)	Suggested structure	Molar conductivity S ⋅ mol^−1^ ⋅ cm^2^ in DMF and DMSO*
	*ν*_1_ 6388 (cal.)	^4^A_2_ → ^4^T_2_		470.2	6112	4.5	Tetrahedral	32.12*
C_1a_	*ν*_2_ 10752	^4^A_2_ → ^4^T_1_ (F)	1.3					
Co(II)	*ν*_3_ 16930 (avr.)	^4^A_2_ → ^4^T_1_ (P)	(0.484)					
	*ν*_4_ 21008	L → M (C.T)						
	*ν*_1_ 6388 (cal.)	^4^A_2_ → ^4^T_2_		470.2	6112	4.61	Tetrahedral	29.7*
C_1b_	*ν*_2_ 10752	^4^A_1_ → ^4^T_1_ (F)	1.3					
Co(II)	*ν*_3_ 16930 (avr.)	^4^A_2_ → ^4^T_1_ (P)	(0.484)					
	*ν*_4_ 21881	L → M (C.T)						
C_2_	*ν*_1_ 12345	^3^A_2_g → ^3^T_2_g	2.8	454.2	12717	3.31	Octahedral	46.45
Ni(II)	*ν*_2_ 16806	^3^A_2_g → ^3^T_1_g (F)	(0.440)					
	*ν*_3_ 27035 (cal.)	^3^A_2_g → ^3^T_1_g (P)						
	*ν*_1_ 12150	^2^B_1_g → ^2^A_1_g				2.36	Octahedral	68.19
C_3_	*ν*_2_ 16666	^2^B_1_g → ^2^B_2_g						
Cu(II)	*ν*_3_ 18761	^2^B_1_g → ^2^Eg						
	*ν*_4_ 19646	L → M (C.T)						
C_4_	*ν*_1_ 12048	^1^A_1_g → ^1^A_2_g				Diamagnetic	Square planar	60.37
Pd(II)	*ν*_2_ 16949	^1^A_1_g → ^1^B_1_g						
	*ν*_3_ 20618	^1^A_1_g → ^1^Eg						
C_5_	*ν*_1_ 17825	^1^A_1_g → ^3^T_1_g (H)				Diamagnetic	Octahedral	154.13
Pt(IV)	*ν*_2_ 22371	^1^A_1_g → ^3^T_2_g						
	*ν*_1_ 6535 (cal.)	^4^A_2_ → ^4^T_2_		487.3	6091	4.21	Tetrahedral	34.3*
C_6a_	*ν*_2_ 10526	^4^A_2_ → ^4^T_1_	1.25					
Co(II)	*ν*_3_ 16666	^4^A_2_ → ^4^T_1_ (P)	(0.501)					
	*ν*_4_ 21551	L → M (C.T)						
	*ν*_1_ 6389 (cal.)	^4^A_2_ → ^4^T_2_		488.5	6107	4.30	Tetrahedral	30.52*
C_6b_	*ν*_2_ 10504	^4^A_2_ → ^4^T_1_ (F)	1.25					
Co(II)	*ν*_3_ 16612	^4^A_2_ → ^4^T_1_ (P)	(0.503)					
	*ν*_4_ 20876	L → M (C.T)						
	*ν*_1_ 5473 (cal.)	^3^T_1_(F) → ^3^T_2_ (F)		721.5	5768	2.73	Tetrahedral	7.9*
C_7_	*ν*_2_ 11074	^3^T_1_ (F) → ^3^A_2_ (F)	0.82					
Ni(II)	*ν*_3_ 15873	^3^T_1_ (F) → ^3^T_1_ (P)	(0.70)					
	*ν*_4_ 18867	L → M (C.T)						
C_8_	*ν*_1_ 13440	^2^B_1_g → ^2^A_1_g				1.84	Square planar	155.8
Cu(II)	*ν*_2_ 19230	^2^B_1_g → ^2^B_2_g						
C_9_	*ν*_1_ 16949	^1^A_1_g → ^1^A_2_g				Diamagnetic	Square planar	125.4
Pd(II)	*ν*_2_ 21367	^1^A_1_g → ^1^B_1_g (C.T)						
C_10_	*ν*_1_ 14388	^1^A_1_g → ^3^T_1_g				Diamagnetic	Octahedral	196.6
Pt(IV)	*ν*_2_ 20576	^1^A_1_g → ^3^T_2_g (C.T)						
C_11a_	*ν*_1_ 6410 (cal.)	^4^A_2_ → ^4^T_2_	1.5	436.8	6552	3.959	Tetrahedral	143.5
Co(II)	*ν*_2_ 10000	^4^A_2_ → ^4^T_1_ (F)	(0.449)					
	*ν*_3_ 15641 (avr.)	^4^A_2_ → ^4^T_1_ (P)						
C_11b_	*ν*_1_ 6410 (cal.)	^4^A_2_ → ^4^T_2_	1.5	436.8	6552	3.997	Tetrahedral	150.6
Co(II)	*ν*_2_ 10000	^4^A_2_ → ^4^T_1_ (F)	(0.449)					
	*ν*_3_ 15641 (avr.)	^4^A_2_ → ^4^T_1_ (P)						
C_12_	*ν*_1_ 4994 (cal.)	^3^T_1_ (F) → ^3^T_2_ (F)	0.74	673.1	4980	2.746	Tetrahedral	159.4
Ni(II)	*ν*_2_ 10482	^3^T_1_ (F) → ^3^A_2_ (F)	(0.653)					
	*ν*_3_ 15483 (avr.)	^3^T_1_ (F) → ^3^T_1_ (P)						
C_13_	*ν*_1_ 12820	^1^A_1_g → ^1^A_2_g				Diamagnetic	Square planar	148.2
Pd(II)	*ν*_2_ 16666	^1^A_1_g → ^1^B_1_g						

**(a):** 

C_1_		
[(L_I_)_2_Co_2_Cl_3_]Cl	Temperature range of decomposition °C	%Weight loss found (calc.)
M ⋅ wt = 841.8		
–2Cl		
–2ph	251–369	41.128 (41.45)
–C_5_H_6_N_2_O_2_		
–OH	370–421	2.798 (2.01)
–2Cl	465–547	7.932 (8.43)
–(C_7_H_3_N_4_OS_4_)2Co		48.25 (48.08)

**(b):** 

C_3_		
[L_I_CuCl(H_2_O)]Cl.0.5BuOH	Temperture range of decomposition °C	%Weight loss found (calc.)
M ⋅ wt = 480.5		
–BuOH	356–476	36.592 (36.94)
–Cl		
–H_2_O		
–CS		
–C_2_H_3_O		
–C_2_NS	477–630	18.008 (17.68)
–NH		
–(phCHNO) CuCl		45.41 (45.36)

**(c):** 

C_4_		
[(L_I_)_2_PdCl]Cl	Temperture range of decomposition °C	%Weight loss found (calc.)
M ⋅ wt = 759		
–2Cl	145–219	14.925 (15.01)
–C_2_H_3_O		
–phC_3_H_4_NO_2_	219–351	21.189 (21.34)
–ph	482–568	10.538 (10.01)
–CN	679–735	3.188 (3.42)
–(C_6_H_3_N_4_OS_4_) Pd		50.096 (50.197)

**(d):** 

C_7_		
[L_II_NiCl2]	Temperture range of decomposition °C	%Weight loss found (calc.)
M ⋅ wt = 468.7		
–CO	50–127	9.183 (9.38)
–NH_2_		
–phCH_2_	239–377	19.672 (19.415)
–2Cl	432–565	34.42 (34.35)
–phN		
–(C_3_NS_2_)Ni		36.858 (36.84)

**Table 4:** Antibacterial and Antifungal activities of studied compounds.

Compounds	*Staphylococcus aureus* inhibition	*Proteus vulgris* inhibition	*Candida albecans* inhibition	*Aspergillus niger* growth
	diameter (mm) 1000 ppm	diameter (mm) 1000 ppm	diameter (mm) 1000 ppm	diameter (mm) 1000 ppm
DMSO	Zero	Zero	Zero	25
Isatin	3	8	6	
N-acetylisatin	4	Zero	5	
N-benzylisatin	5	5	6	
N-benzoylisatin	5	5	Zero	
L_I_	8	5	Zero	9
C_2_ (Ni(II))	4	8	Zero	
C_3_ (Cu(II))	9	5	5	
C_4_ (Pd(II))	18	5	Zero	9
L_III_	6	8	Zero	9
C_11_ (Co(II))	3	10	14	9
C_12_ (Ni(II))	Zero	12	11	
C_13_ (Pd(II))	3	Zero	11	
